# The Role of gp130 Cytokines in Tuberculosis

**DOI:** 10.3390/cells9122695

**Published:** 2020-12-15

**Authors:** Kristina Ritter, Jasmin Rousseau, Christoph Hölscher

**Affiliations:** 1 Infection Immunology, Research Centre Borstel, D-23845 Borstel, Germany; kritter@fz-borstel.de (K.R.); jrousseau@fz-borstel.de (J.R.); 2 German Centre for Infection Research (DZIF), Partner Site Hamburg-Borstel-Lübeck-Riems, D-23845 Borstel, Germany

**Keywords:** tuberculosis, cytokines, gp130, IL-6, IL-11, IL-27

## Abstract

Protective immune responses to *Mycobacterium tuberculosis* (Mtb) infection substantially depend on a delicate balance within cytokine networks. Thus, immunosuppressive therapy by cytokine blockers, as successfully used in the management of various chronic inflammatory diseases, is often connected with an increased risk for tuberculosis (TB) reactivation. Hence, identification of alternative therapeutics which allow the treatment of inflammatory diseases without compromising anti-mycobacterial immunity remains an important issue. On the other hand, in the context of novel therapeutic approaches for the management of TB, host-directed adjunct therapies, which combine administration of antibiotics with immunomodulatory drugs, play an increasingly important role, particularly to reduce the duration of treatment. In both respects, cytokines/cytokine receptors related to the common receptor subunit gp130 may serve as promising target candidates. Within the gp130 cytokine family, interleukin (IL)-6, IL-11 and IL-27 are most explored in the context of TB. This review summarizes the differential roles of these cytokines in protection and immunopathology during Mtb infection and discusses potential therapeutic implementations with respect to the aforementioned approaches.

## 1. Introduction

Tuberculosis (TB) is still the leading cause of death from an infectious agent and thus represents a major health problem, with one quarter of the global population infected with *Mycobacterium tuberculosis* (Mtb) [1]. In 2018, about 1.5 million people died from TB and nearly 10 million people worldwide fell ill with TB [1]. Infection with Mtb is mainly initiated by aerogenic exposure to a patient with active pulmonary TB [2]. Inhaled bacteria are quickly phagocytized by alveolar macrophages and a small granulomatous lesion, containing neutrophils, macrophages, multinucleated giant cells and lymphocytes, develops which in most cases prevents the systemic spread and limits the growth of Mtb [2,3]. The majority of infected individuals (>90%) remain latently infected without developing any symptoms. However, because containment of Mtb in these individuals is facilitated by an active immune response, anti-inflammatory therapies to treat autoimmune and chronic inflammatory diseases such as rheumatoid arthritis, psoriasis and Crohn’s disease increase the risk of reactivation of latent TB [4,5,6,7]. Eventually, 5 to 10% of infected individuals develop active TB caused by reactivation of latent TB accompanied by chronic inflammation [3,8]. These active TB patients require at least 6 months of treatment with multiple drugs, but the spread of multi-drug resistant (MDR-TB) and extremely drug-resistant (XDR-TB) strains has made the management of TB more challenging because of the poor, expensive, less-effective and toxic alternatives to the first-line drugs [9,10]. New treatment regimens interconnecting TB drugs and immunomodulation as adjunct therapy (host-directed therapy, HDT) may help to shorten the treatment duration and thereby prevent the development of drug resistant Mtb [8,9,11]. In order to develop novel immunomodulatory interventions (1) for the anti-inflammatory therapy of potentially latently Mtb-infected patients suffering from autoimmune or chronic inflammatory diseases or (2) for the adjunct treatment of TB, the understanding of the mechanisms that mediate protection but also pathogenesis in TB is mandatory.

**Figure 1:**
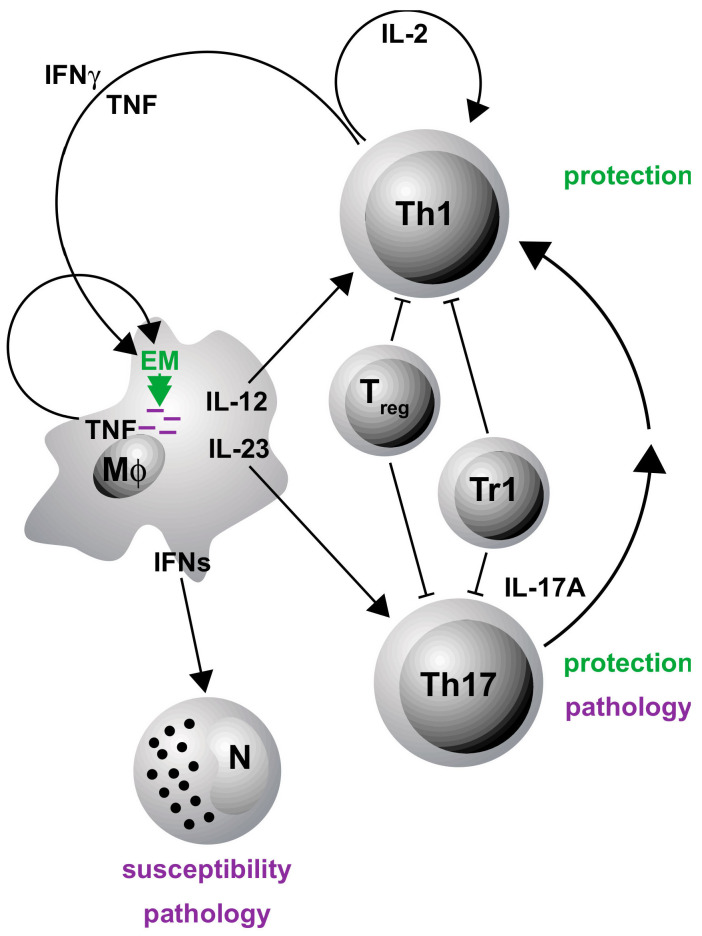
The immune response to Mtb infection. After phagocytosis of Mtb (pink rods) by macrophages (Mϕ), cytokines such as TNF, IL-12 and IL-23 are released. IL-12 is critically important for the induction of TH1 cells, whereas IL-23 mediates the differentiation of IL-17A-producing TH17 cells. By activating various chemokines, IL-17A indirectly contributes to granuloma formation and the recruitment of IFNγ/TNF/IL-2-producing multifunctional T cells to the site of Mtb infection. IFNγ and TNF in turn synergistically activate effector mechanisms (EM) in infected Mϕ. Through this activation cascade, TH17 and TH1 cells mediate protection against Mtb infection. However, an elevated TH17 immune response can also have pathological consequences. Susceptibility to and the subsequent pathology of tuberculosis (TB) are mediated by the activity of neutrophils dependent on type 1 interferons (IFNs). T_reg_ and Tr1 cells accumulate at the site of infection and restrict protective T cell responses.

Dysregulated secretion of cytokines or the lack of cytokines/cytokine receptors and their subsequent signaling pathways contribute to susceptibility and/or pathogenesis of infectious diseases in humans and various animal models [12,13,14]. In this context, cytokines were shown to be in host defense against Mtb by supporting a cellular immune response required for the control of mycobacterial growth [15,16] but also prevent a detrimental inflammatory immune response [17,18]. A type 1 or T helper 1 (TH1) immune response is instructed by the stimulation of naïve CD4^+^ T cells through interaction with antigen-presenting cells (APCs) that express cytokines, costimulatory molecules and other polarizing signals that promote the differentiation into effector TH1 cells [15,19]. In particular, interleukin (IL)-12, produced after phagocytosis of Mtb by macrophages and dendritic cells (DC), is needed for the induction of TH1 cells (Figure 1). These cells typically secret interferon (IFN)γ and tumor necrosis factor (TNF), leading to a synergistic activation of anti-mycobacterial effector mechanisms in macrophages [20,21] (Figure 1) and an elevated production of pro-inflammatory cytokines such as IL-1β, IL-6 and TNF [12,22,23].

In recent decades, IFNγ-producing CD4^+^ T cells were considered to be the main arm of a protective cellular immune response by conveying granuloma organization and bacterial killing of macrophages in TB patients and animal models of TB [24,25,26]. However, this view is currently under debate due to a poor correlation between the levels of IFNγ and the degree of protection against the infection [27,28,29]. In addition to the IFNγ-dominated TH1 immune response, another cell population, IL-17A-producing TH17 cells, came into focus that supports protection against Mtb [27,28,30,31] (Figure 1).

TH17 cells differentiate from naïve T cells through the interaction with various cytokines such as IL-6 and are maintained by IL-23 [32]. In TB, IL-17A contributes indirectly to granuloma formation and the recruitment of IFNγ/TNF/IL-2-producing multifunctional T cells to the site of infection by the induction of various chemokines [17,33,34] (Figure 1). However, because TH1 and TH17 cells are both strong inducers of inflammatory immune responses, both also contribute to chronic inflammation and tissue destruction in experimental Mtb infection [17,18] (Figure 1). Hence, a balanced activation of TH1 and TH17 cells is needed to restrict mycobacterial growth and to limit immunopathology [28,31,35]. Checkpoints of these TH1 and TH17 immune responses are regulatory T cells (T_reg_) and type 1 regulatory (Tr1) cells, which accumulate in the lungs at an early stage of the infection and thus delay the migration of effector T cells [36,37]. However, it can also be assumed that these regulatory cells keep a pathological inflammatory reaction in check in the course of TB (Figure 1). While TH1 and TH17 cells are rather implicated in protective immune responses, susceptibility to TB and the pathology of this disease are facilitated by the activity of neutrophils which strikingly depend on type 1 interferons [38] (Figure 1). In respect to novel adjunct therapeutic approaches for the management of active TB, immunomodulation has to promote protective immune responses without the risk of developing a pathological inflammation.

In addition to IFNγ and IL-17A, TNF also plays a crucial role in the containment of Mtb infection by sustaining the granuloma structural integrity and promoting anti-mycobacterial effector mechanisms in macrophages [2,12,39]. Accordingly, anti-TNF immunotherapy, which is used to treat autoimmune and chronic inflammatory diseases, disrupts effective immunity against Mtb and eventually increases the risk of reactivation of latent TB [4,5,6,7]. TNF blockers are therefore an important example of how immunomodulatory therapies may adversely affect host immunity in TB. The development of novel anti-inflammatory treatment strategies for autoimmune or chronic inflammatory diseases must always take the risk of reactivation of latent TB into account.

Altogether, cytokine-directed interventions in the host immune system may open two major perspectives to advance prevention and control of TB: the identification of improved approaches to treat inflammatory diseases without compromising anti-mycobacterial immunity and the development of new therapeutic strategies in the treatment of TB, which especially include HDTs. In both respects, cytokines/cytokine receptors which are linked to the signal-transducing receptor gp130 may serve as promising target candidates. This review therefore summarizes the role of the gp130-related cytokines in the context of Mtb infection and discusses potential therapeutic implementations of these cytokines with regard to the aforementioned approaches.

## 2. gp130-Related Cytokines

The gp130 cytokine receptor family is defined by shared structural features of both ligands and receptors. These receptors are utilized by a variety of functionally and structurally related cytokines within the IL-6 and IL-12 family including IL-6, IL-11, IL-27, IL-35, IL-39, leukemia inhibitory factor (LIF), oncostatin M (OSM), ciliary neurotrophic factor (CNTF), cardiotrophin-1 (CT-1), novel neutrophin-1/B cell stimulating factor-3 or cardiotrophin-like cytokine (CLC) and neuropoetin (NP) [40,41,42,43,44,45,46,47,48,49,50,51,52,53] (Table 1). Structurally related to the IL-6 cytokine family is the newly discovered cytokine IL-31 [54]. This cytokine is gp130-independent but utilizes the ortholog of gp130, IL-31Rα chain and OSM receptor β (OSMR) [54,55,56].

The subunit gp130 is a common receptor component belonging to the type I cytokine receptor family. The shared usage of gp130 within the gp130 cytokine receptor family results in functional redundancy [52,57]. While gp130 is ubiquitously expressed, specificity and distinctness of action can be attributed to the use of ligand-specific receptor components that show a more limited expression pattern [52,58,59]. All members of the gp130 cytokine family induce Janus Kinase/Signal Transducer and Activator of Transcription (JAK/STAT) molecules, mostly STAT3 but also, to a lesser extent, STAT1 [60]. Alternatively, also other signal transduction pathways can be activated, however, in all receptor complexes, gp130 is required for signaling. In TB, IL-6, IL-11 and IL-27 are the most explored gp130 cytokines and should therefore be discussed in this review.

### 2.1. IL-6

IL-6 is the representative member of the IL-6 cytokine family. It has a four a-helix bundle structure [61] and, initially, many names were attributed to this cytokine due to its diverse functions, such as B cell stimulatory factor 2 [62], hybridoma growth factor [63], plasmacytomas growth factor [64], hepatocyte-stimulatory factor [65] and cytotoxic T cell differentiation factor [66], until it became clear that all of these properties were attributable to a common cytokine. The names already indicate its broad-ranging effects in hematopoiesis, tissue homeostasis, metabolism, neurogenesis and immunology. IL-6-mediated immune mechanisms include, inter alia, the induction of various acute phase proteins, the induction of immunoglobulin production in B cells and the proliferation and differentiation of T lymphocytes [52,67,68]. After infection, in essence, IL-6 is produced by many cell types of which the most important are monocytes and macrophages and is believed to play a central role in hosts defense mechanisms against various infectious agents [69,70,71].

**Figure 2:**
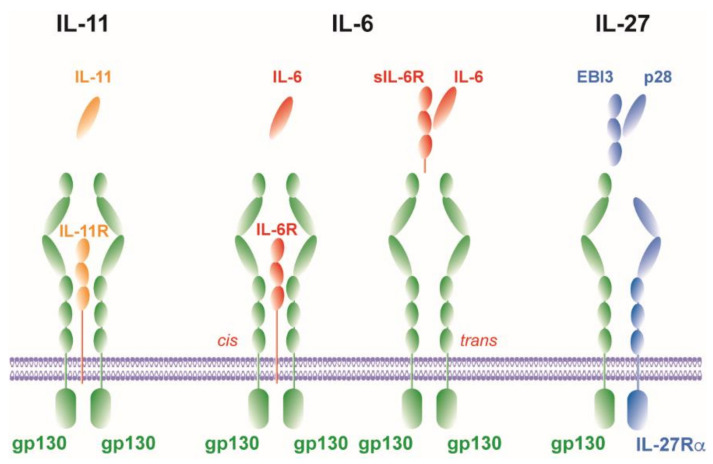
Ligands and receptor complexes of the gp130 cytokines IL-6, sIL-6R/IL-6, IL-11 and IL-27. The gp130 cytokines IL-6 and IL-11 represent monomeric four-helix bundle cytokines. In contrast, sIL-6R/IL-6 and IL-27 are heterodimeric cytokines. Both comprise a secreted receptor α subunit and a four-helix bundle protein (sIL-6R/IL-6 and EBI3/IL-27p28, respectively). These cytokines all signal through receptor complexes that contain the gp130. The receptor complexes for IL-6 and IL-11 comprise a non-signaling receptor α subunit and a signaling gp130 homodimer. IL-6 signals through this receptor complex in *cis*. In contrast, a gp130 homodimer is required for sIL-6R/IL-6-mediated signaling. Accordingly, the receptor complex for IL-27 contains gp130 and the IL-27R subunit-α, which is structurally similar to gp130.

It is now known that IL-6 mediates different mechanisms via two fundamentally different pathways. It can signal via a membrane-bound receptor (*cis*) or, after proteolytic cleavage of the IL-6 receptor (R) from the cell membrane, via a soluble receptor (*trans*) (Figure 2) [72].

#### 2.1.1. Cis-Signaling

In *cis*-signaling—also called *classical* signaling—IL-6 binds to the membrane-bound IL-6Rα chain (mIL-6Rα) (Figure 2). The complex consisting of IL-6/mIL-6Rα recruits homodimers of gp130 which triggers a downstream JAK/STAT signaling cascade [68,73] (Figure 2). Consequently, because gp130 is universally expressed, *cis*-signaling is only relevant for cells that express mIL-6Rα (i.e., hepatocytes, neutrophils, monocytes/macrophages and CD4^+^ T cells [65,72]). By using IL-6-deficient (^-/-^) mice, IL-6 has been shown in the 1990s to mediate the acute phase response during inflammation [74] and to promote TH1 immune responses [75]. More recently, IL-6 *cis*-signaling acts in a pro-inflammatory fashion by tipping the balance from the development of T_reg_ towards a TH17 immune response. IL-6 favors the differentiation of IL-17A-producing TH17 cells and inhibits the generation of T_reg_ [68,76,77,78,79]. Since dysregulation of the TH17/T_reg_ balance is linked to the development of autoimmunity and chronic inflammatory diseases [80,81], the induction of a TH17-type of inflammation characterizes a major pro-inflammatory function of IL-6 *cis*-signaling.

In contrast to the pro-inflammatory role of IL-6 *cis*-signaling in direct interaction with CD4^+^ T cells in TH1 immune responses and during the development of TH17 cells, IL-6 can also act in an anti-inflammatory fashion through the mIL-6Rα on macrophages because IL-6 *cis*-signaling inhibits the release of pro-inflammatory cytokines such as IL-12 and IL-23 by activated macrophages in vitro [82,83] and is able to promote alternative macrophage activation during obesity-related inflammation in mice leading to an ameliorated disease outcome [84].

Hence, IL-6 *cis*-signaling can act in a pro-inflammatory as well as in an anti-inflammatory fashion apparently depending on the type of target cell.

#### 2.1.2. Trans-Signaling

In addition to *cis*-signaling, there is an alternative signaling pathway called *trans*-signaling. Here, IL-6 binds to a soluble IL-6Rα chain (sIL-6Rα) that is secreted after proteolytic cleavage of mIL-6Rα by the metalloprotease ADAM17 in a process called shedding [85,86] (Figure 2). *Trans*-signaling can evoke downstream signaling in cells, which do not express mIL-6Rα and, as a result, do not respond to IL-6, by binding of the IL-6/sIL-6Rα complex to the ubiquitously expressed gp130 subunit [87] (Figure 1). The development of a fusion protein of sIL-6Rα and IL-6, linked by a flexible peptide chain, called hyper-IL-6 allowed discriminating between *trans*-signaling and *cis*-signaling [88]. Binding of IL-6/sIL-6Rα promotes mainly pro-inflammatory immune responses characterized by the recruitment of immune cells [89,90], prevention of apoptosis [91] and inhibition of T_reg_ differentiation [92]. The naturally occurring sgp130 serves as a potent antagonist of IL-6 *trans*-signaling [93] and selective inhibition of IL-6 *trans*-signaling by recombinant sgp130 fused to the Fc region of human IgG1 (sgp130Fc) suppresses a detrimental outcome of many inflammatory diseases [91,94,95,96,97,98,99]. Hence, treatment of autoimmune and chronic inflammatory disorders with sgp130Fc represents a valuable anti-inflammatory therapeutic strategy.

#### 2.1.3. Trans-Presentation

Recently, another mode of IL-6 signaling called *trans*-presentation was described [100]. In this condition, in the DC–T cells interaction zone, the IL-6/mIL-6Rα complex on DCs is presented to gp130 expressed on T cells. This *trans*-presentation stimulates the STAT3-dependent development of pathogenic TH17 cells [100].

### 2.2. IL-11

The cytokine IL-11 belongs to the IL-6 cytokine family and is a monomeric cytokine. IL-11-induced signaling is mediated by the formation of the receptor complex, composed of the ligand-binding subunit IL-11Rα and the β-subunit gp130 (Figure 2). The α subunit of the receptor is expressed in lymphocytes, B cells, macrophages, endothelial cells, hematopoietic cells and osteoclasts [101]. Due to this abundant expression, IL-11 exerts pleiotropic effects such as the stimulation of hemopoiesis [102], thrombopoiesis [103] and the modulation of macrophage differentiation [104].

In several models of inflammation and infection, IL-11 was identified to be an immunomodulatory cytokine as it inhibits the release of pro-inflammatory cytokines [105,106,107,108]. However, also pro-inflammatory properties have been attributed to the cytokine. Thus, overexpression of IL-11 drives lymphocytic inflammation and fibrotic tissue remodeling in the murine airways [109] as well as heart and kidney fibrosis in mice [110]. Moreover, IL-11 is also involved in the recruitment of neutrophils to sites of inflammation or infection [111,112]. Most recently, IL-11 was demonstrated to promote the differentiation of TH17 cells in patients with multiple sclerosis (MS) and during relapsing-remitting experimental autoimmune encephalomyelitis (RREAE) [112]. Similar to IL-6, *trans*-signaling has also been described for IL-11 [113,114] (Figure 2). However, no biological function has been ascribed to IL-11 *trans*-signaling yet.

### 2.3. IL-27

In contrast to the IL-6 cytokine family members IL-6 and IL-11, the IL-12 cytokine family member IL-27 forms a heterodimeric complex [115] (Figure 2). The IL-12 family α subunit IL-27p28 resembles the unique up-up-down-down four-α-helix bundle structure of the IL-6 family made from a single polypeptide [12,116,117], which pairs with the β subunit Epstein-Barr-virus-induced gene 3 (EBI3) that is structurally related to sIL-6Rα [115,118]. The heterodimeric structure of IL-27 is analogous to complexes composed of soluble receptors such as sIL-6Rα that dimerize with their corresponding ligands [60]. IL-27 is produced by APCs in response to Toll-like receptor (TLR) activation [119,120]. It signals through a heterodimeric receptor complex composed of the private ligand-binding IL-27Rα and the common gp130 subunit [48] (Figure 2). IL-27R is expressed on various types of immune cells (e.g., T cells, macrophages, DC). Initially, IL-27 was shown to represent a key pro-inflammatory mediator of TH1 polarization by inducing the transcription factor T-bet and subsequently the expression of the IL-12 receptor β2 chain in naïve CD4^+^ cells. Hereby, IL-27 instructs TH0 cells to respond to IL-12 to differentiate into TH1 cells [119,121]. Hence, IL-27 instructs the initial TH1 response [122,123]. In contrast to this pro-inflammatory function during the initiation of a cell-mediated immune response, IL-27 induces a broad spectrum of suppressive mechanisms under inflammatory conditions. In general, it limits the intensity and duration of TH1, TH2 and TH17 cell activity and their cytokine production. Under strongly polarized TH1 immune responses, IL-27 suppresses the hyperactivity of CD4^+^ T cells and the development of inflammatory diseases [18,124]. Under TH2 conditions, IL-27 downregulates the TH2 transcription factor GATA3 [123,125]. Additionally, IL-27 also negatively regulates the differentiation of TH17 cells by several mechanisms, including the control of IL-6 production [126] (that, together with transforming growth factor-beta (TGF-β), supports the development of TH17 cells [32]) and of the TH17-specific transcription factor retinoid-related orphan receptor gamma t (RORγt) [127]. In line with this anti-inflammatory function just described, IL-27 also promotes the function of T_reg_ under inflammatory conditions [128], which are central regulators of cellular immune responses and limit excessive inflammation. IL-27 upregulates in T_reg_-suppressive molecules such as LAG3, but the precise mechanisms by which IL-27 controls T_reg_ functions remain elusive [128,129]. Additionally, IL-27 has been recognized as a differentiation factor for IL-10-producing Tr1 cells, which are crucial in controlling tissue inflammation [130] by inducing c-Maf, a transcription factor that transactivates IL-21 secretion which acts as an autocrine growth factor for Tr1 cells [131,132].

Furthermore, at the level of APCs, IL-27 is able to limit the release of pro-inflammatory cytokines and induce immunoregulatory molecules such as CD39 in activated macrophages and DCs, respectively. Therefore, IL-27 can also indirectly contribute to the modulation of TH1 and TH17 immune responses by suppressing the release of the TH1- and TH17-driving cytokines IL-12 and IL-23 in accessory cells, respectively [18,133,134].

## 3. gp130 Cytokines in Tuberculosis

This review aims to summarize the differential roles of gp130 cytokines in TB. Thereby, the main focus is on the presentation of mouse experimental data on the impact of the cytokines IL-6, IL-11 and IL-27, which are most explored in the context of TB. Moreover, the current study situation of these cytokines during human TB is delineated and potential therapeutic implementations are discussed. The animal experiments referred to here mainly deal with the impact of these mediators on TH1 and TH17 immune responses after infection with Mtb. Even if gp130 cytokines have pleiotropic effects, e.g., on TH2, cytotoxic T or B cells, these have only been little researched in connection with the role of IL-6, IL-11 and IL-27 in experimental TB. Although this review does not deal with these arms of the Mtb-induced immune response in the context of gp130 cytokines, future studies are certainly important.

The pleiotropic modes of action, along with the shared usage of both cytokine and receptor subunits within the gp130 cytokine family [52,116], imply a challenge for the experimental investigation of these cytokines during Mtb infection. Therefore, consideration of complementary mouse models, which interfere with the gp130-dependent signaling cascade at different levels, is of particular importance. Mice with a global cytokine deficiency can provide information on the overall influence of a certain cytokine on the outcome of Mtb infection, however, these mouse models carry the risk of concealing TB-related pleiotropic effector mechanisms. Mouse models, which cell-specifically interfere with gp130-mediated signaling, represent an interesting tool to highlight such effects. When interpreting findings obtained from these mouse models however, it has to be considered that the observable effects cannot necessarily be traced back to a specific cytokine. In addition to the choice of animal model, experimental parameters such as the bacterial dose and the route of exposure appear to have a considerable impact on the experimental outcome [135,136]. Likewise, the impact of specific cytokines on the outcome of experimental TB may also be influenced by the use of a more virulent Mtb strain or clinical isolate. Together, mouse experimental data on the impact of gp130 family cytokines during TB indeed provide important insights, however, the described limitations of these models may result in discrepancies in findings obtained from human studies.

### 3.1. IL-6

Although the cellular sources of IL-6 are known in principle, they have not been adequately investigated in either Mtb-infected mice or TB patients. Only in the context of murine type 2 diabetes was IL-6 described to be produced after infection by natural killer cells and CD11c DC [137]. Initially, IL-6 was considered a pro-inflammatory cytokine involved in protection against Mtb. However, during experimental TB, IL-6 plays a more complex role which differs between experimental models and, in addition, appears to be dependent on experimental conditions such as the infectious dose and the route of exposure. Accordingly, IL-6^-/-^ mice are susceptible to a systemic infection with high doses of intravenously (i.v.) delivered Mtb [135]. The absence of IL-6 here leads to increased IL-4 levels and an attenuated expression of IFNγ. Moreover, the antibody-mediated depletion of IL-6 upon i.v. infection with *Mycobacterium avium* enhances mycobacterial growth [138]. In contrast, after aerosol infection with Mtb, IL-6^-/-^ mice exhibit only an initial increase in bacterial burdens accompanied by a delayed IFNγ induction; however, these mice are eventually able to contain mycobacterial growth and to mount a protective memory response to secondary infection [136]. Moreover, frequencies of TH17 cells as well as of T_reg_ are also only slightly modified in Mtb-infected IL-6^-/-^ mice [139] (submitted). Together, different from the indispensable role of cytokines such as IFNγ and TNF during experimental TB, IL-6 may only be involved—either directly or indirectly—in early protective immune responses, but overall, effective anti-mycobacterial protection appears to be only marginally dependent on IL-6.

**Figure 3:**
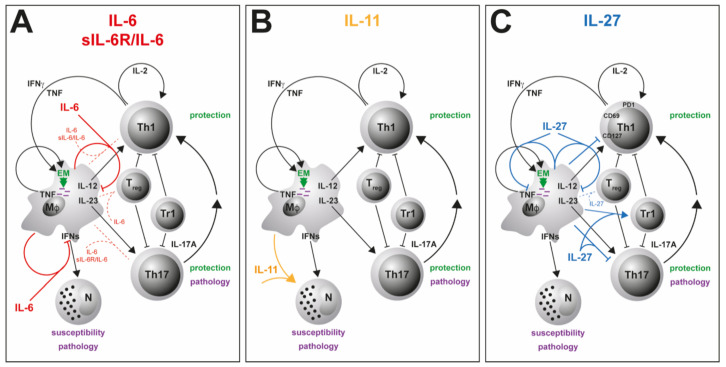
The effect of gp130 cytokines on the immune response to an infection with Mtb. (**A**) While IL-6 and sIL-6R/IL-6 do not have any effect on TH1, TH17 and T_reg_, IL-6 is able to restrict the production of IL-12, IL-23 and type 1 interferons (IFNs) in activated macrophages. This can indirectly influence the activation of neutrophils and T cells but has no influence on the outcome of the infection. (**B**) IL-11 promotes the accumulation of pathogenic neutrophils and could thereby contribute to TB pathology. (**C**) IL-27 suppresses the parenchymal accumulation of PD-1-, CD69- and CD127-expressing self-renewing TH1 cells and, by limiting the expansion of IL-17A-producing TH17 cells, the recruitment of IFNγ/TNF/IL-2-producing multifunctional TH1 cells. TH1 and TH17 immune responses may also be indirectly diminished by IL-27 through the activation of Tr1 cells rather than of T_reg_. Importantly, IL-27 also has a suppressive effect on macrophage effector functions (EM) and on the release of pro-inflammatory cytokines by these cells. Even if IL-27 suppresses protective functions, it also prevents immunopathological consequences of an infection with Mtb by controlling an excessively strong TH17 immune response.

Due to the pleiotropy of IL-6, a more precise picture of its role during Mtb infection is presented by the cell type-specific analysis of IL-6/gp130-mediated effects. The CD4^+^ T cell-specific deficiency of gp130—and thus of IL-6-mediated signaling on T cells—leads to the abrogation of TH1 and TH17 immune responses in mouse models of autoimmunity [79] or extracellular parasitic infection [140]. Analysis of CD4^+^ T cell-specific gp130-deficient mice during Mtb infection, however, revealed that, here, not only the frequency of TH1 cells, but also the induction of TH17 cells and the overall expression of *IL17a* appear to be largely independent of gp130 expression on T cells [139] (submitted) (Figure 3A). In light of the repeatedly described impact of IL-6-mediated signaling on TH17 differentiation [32,79,140], this finding seems rather surprising. A compensatory effect of IL-27-mediated signaling on TH17 development, which is also abrogated in T cell-specific gp130-deficient mice, however, may partly explain this effect as the expansion of TH17 cells is only partly reduced in Mtb-infected IL-6^-/-^ mice [139] (submitted). Hence, in contrast to IL-23 [33], IL-6 appears not to be required for a robust TH17 immune response during experimental TB. In accordance with the unaffected pro-inflammatory immune response, deficiency of gp130 on T cells results in only moderately decreased bacterial loads during the course of Mtb infection [139] (submitted).

Despite its pro-inflammatory properties, IL-6 appears to exert suppressive effects in macrophages [83,141]. Accordingly, IL-6 produced by Mtb-infected macrophages limits the responsiveness of uninfected macrophages to IFNγ [142], indicating that IL-6 also mediates anti-inflammatory mechanisms in TB (Table 2). In this context, IL-6 suppresses the transcription of selective IFNγ-responsive genes in infected macrophages [142]. An immunosuppressive role of IL-6 during mycobacterial infection was confirmed by VanHeyningen et al. [143] (Figure 3A; Table 2). Here, macrophages display a decreased T cell activation capacity in response to infection with *Mycobacterium bovis* BCG (Bacillus Calmette–Guérin), which is reversed by preincubation with neutralizing antibodies against IL-6. This cell type-specific effect has been further shown in vivo by using macrophage/neutrophil-specific gp130-deficient mice [82]. These mice exhibit elevated levels of pro-inflammatory cytokines and enhanced TH1 and TH17 immune responses, together with an increased expression of the anti-mycobacterial effector molecules inducible nitric oxide synthase (*Nos2*) and *Lrg47* (EM in Figure 3A; Table 2). Nevertheless, the augmented inflammatory immune response in macrophage/neutrophil-specific gp130-deficient mice appears not to be critical for controlling mycobacterial growth. When assessing these data, it certainly has to be taken into account that the specific lack of gp130 in macrophages interferes not only with IL-6-, but also with IL-27-mediated signaling.

Notably, IL-6 also suppresses the expression of type I IFN-related genes in murine macrophages infected with Mtb strains of different virulence [144]. As it was demonstrated that type I IFNs–which are crucially important in the defense against viral infections—play a harmful role during mycobacterial infection [145,146,147], the inhibition of type I IFN-related genes by macrophage-produced IL-6 may contribute to the containment of TB progression [144] (Figure 3A).

A major physiological regulator of IL-6-mediated signaling is the Suppressor of cytokine signaling 3 (SOCS3) [148,149]. SOCS3 acts by inhibiting STAT3-mediated signaling by binding to gp130 [150]. Expression of *Socs3* is induced in Mtb-infected macrophages through MyD88-dependent mechanisms [151] and, during experimental TB, in the lungs of Mtb-infected mice [152]. Mice with a T cell-specific lack of SOCS3 are highly susceptible to experimental TB [151]. Containment of Mtb infection is, however, also dramatically reduced in mice with SOCS3-deficient macrophages [151,153]. Both mouse models display increased levels of *Il6*-expression, which is, however, neither attributable to macrophages nor to T cells. In the absence of macrophage-derived SOCS3, IL-6 appears to contribute to susceptibility to Mtb infection by suppressing IL-12/23p40 secretion in macrophages and the subsequent impairment of TH1 immune responses [151]. However, IL-6 may also directly affect anti-mycobacterial effector mechanisms in Mtb-infected macrophages in the absence of SOCS3. Accordingly, in vitro data revealed that SOCS3 prevents the development of alternatively activated macrophages (AAM), while IL-6 induces the expression of the AAM marker arginase 1 (Arg1) in SOCS3-deficient macrophages [154,155]. During mycobacterial infection, Arg1 derived from AAM counteracts protective macrophage effector mechanisms [152,156,157]. In Mtb-infected mice with a macrophage-specific lack of SOCS3, depletion of IL-6 results in a reduced expression and activity of Arg1 rather than an impaired TH1 immune response accompanied by decreased bacterial loads and less lung pathology [153]. In total, SOCS3 appears to keep IL-6-dependent macrophage responses under control and therewith preserves protective macrophage effector mechanisms.

In humans, increased expression of IL-6 was found in the blood of patients with pulmonary TB when compared to a healthy control group [158,159]. Moreover, the diagnostic value of IL-6 was higher in tuberculous pleural effusion when compared to malignant effusion [160,161]. On the other hand, a preliminary multiplex analysis further revealed a significantly higher IL-6 secretion in latently Mtb-infected patients than in patients with active TB [162]. However, later studies did not confirm any difference in IL-6 secretion levels between latent and active TB patients [163] or between latently and active Mtb-infected groups and non-TB patients [164]. Conversely, patients with cavitary TB—a severe form of pulmonary disease—were found to exhibit a reduced content of IL-6 in the bronchoalveolar lavage (BAL) in comparison to TB patients without cavities, suggesting the cytokine as a potential biomarker for protection against tissue destruction during advanced TB [165]. Recently, the association of four single-nucleotide polymorphisms (SNPs) in the IL-6 gene with TB susceptibility was explored in the Western Chinese Han population [166]. However, a link between susceptibility to TB and IL-6 polymorphisms was not identified within the scope of this study.

Overall, the conflicting data regarding the role of IL-6 in the context of mycobacterial infection are not surprising, but rather reflect the pleiotropic and multifunctional nature of the cytokine [167,168]. During experimental TB, IL-6 appears to interfere with innate as well as adaptive immunity and to mediate pro-inflammatory as well as immunosuppressive effector responses [82,135,136,142,151,153]. Accordingly, depending on the experimental model and most importantly on the target cell, IL-6 has a protective or detrimental effect on the progression of TB [82,135,136,139,153]. The gp130-binding suppressor molecule SOCS3 may therefore be critical in specifically controlling the IL-6-mediated development of Mtb-permissive macrophages [153]. Together, counteraction of the IL-6-mediated pro- and anti-inflammatory effects along with the SOCS3-dependent regulation of IL-6 activity may account for the largely unaffected outcome of experimental TB in global IL-6^-/-^ mice. Nevertheless, it can be stated that IL-6 plays a rather subordinate role in the protective immune response against Mtb (Table 2). Surprisingly, IL-6-mediated signaling also appears to be almost negligible for TH17 differentiation during TB [139] (submitted).

### 3.2. IL-6/sIL-6Rα

To fully consider the impact of IL-6 during Mtb infection, the specific role of IL-6/sIL-6Rα *trans*-signaling for the outcome of disease also needs to be addressed. As aforementioned, inhibition of the IL-6 *trans*-signaling mechanism—without interfering with the classical IL-6 *cis-*signaling pathway or with IL-6 *trans*-presentation—can be achieved by use of sgp130Fc [93,94,100]. With regard to experimental TB, administration of sgp130Fc to mice during the acute or chronic phase of Mtb infection has no significant effect on the expression of the effector cytokines TNF, IL-6, IFNγ and IL-17A, whereas it slightly hampers the release of IL-12/23p40 [169] (Figure 3A). At the same time, containment of Mtb infection is not impaired by treating infected mice with sgp130Fc. Permanent inhibition of IL-6/sIL-6Rα *trans*-signaling in sgp130Fc-overexpressing (sgp130Fc^tg^) mice results in an inflammatory defect accompanied by reduced infiltration of neutrophils and monocytic cells during acute inflammation [95]. Accordingly, Mtb-infected sgp130Fc^tg^ mice exhibit a timely restricted increase in bacterial burdens in the acute phase of TB, however, overall, these mice also remain able to mount protective immune responses to infection [93]. In conclusion, these data clearly demonstrate that IL-6/sIL-6Rα *trans*-signaling is dispensable for anti-mycobacterial immunity and containment of Mtb (Table 2).

### 3.3. IL-11

Hitherto, the role of the IL-6 family member IL-11 during Mtb infection has barely been investigated. Research conducted by Alexander S. Apt and colleagues, however, suggests the cytokine as a potential host therapeutic target for immune modulatory treatment of TB [170,171,176]. They initially found that IL-11 is strongly expressed by interstitial lung macrophages [176]. Thereby, macrophages from I/St mice, which are susceptible to Mtb infection, exhibit substantially higher expression levels of IL-11 than those from resistant A/Sn mice. Importantly, because, after infection of the genetically heterogeneous F2 progeny of the I/St and A/Sn strains with Mtb, the individual expression levels of IL-11 mRNA in the lung tissue correlate with the degree of disease-associated body weight loss, a potential role of this cytokine as a risk factor for TB progression can reasonably be assumed [177]. Eventually, the antibody-mediated depletion of IL-11 in Mtb-infected susceptible I/St mice diminishes the extent of disease progression [170]. Thereby, in addition to reduced bacterial burdens, the blockade of IL-11 reduces histopathology and the infiltration of the lung tissue by neutrophils. Moreover, the antibody treatment decreases the protein levels of other inflammatory cytokines such as TNF and IL-6. As it also downregulates mRNA expression of *Il11* itself, the authors presume a positive feedback loop at the transcriptional level. Together, these data imply that the self-reinforcing hyperproduction of IL-11 plays a causative role in lung pathogenesis during the early phase of experimental TB in genetically susceptible mice.

To investigate the potential of IL-11-directed therapy for immune modulatory treatment of TB, a recombinant mutated form of IL-11 was established and administered by aerosol in the lungs of Mtb-infected mice [171]. This recombinant IL-11 variant acts as a high-affinity antagonist of IL-11-mediated signaling by competitive disruption of the gp130/IL11R signaling complex formation [178]. Local administration of an IL-11 antagonist takes account for the involvement of IL-11 in several physiological pathways [171,179]. A systemic blockade of IL-11, on the contrary, bears the risk of serious adverse effects [171]. In response to therapy, Mtb-infected mice exhibit substantially reduced levels of TB-associated inflammation in the lung, which includes lower numbers of F4/80^+^ macrophages and Ly-6G^+^ neutrophils in the early phase of Mtb infection and a decreased pulmonary infiltration of lymphocytes during the further course of infection [171] (Table 2). Moreover, treatment decreases the expression of key inflammatory factors such as TNF and IFNγ at both mRNA and protein levels. Likewise, treated mice show lower expression levels of IL-6 and IL-11 itself. Eventually, IL-11-directed therapy prolongs the survival of Mtb-infected animals. Based on these data and the aforementioned findings obtained after systemic administration of anti–IL-11 antibodies, the authors claim that blockade of IL-11 may mediate protection during infection with Mtb particularly by attenuating pathogenic neutrophil accumulation in the affected lung tissue [170,171] (Figure 3B; Table 2). Hence, during experimental TB, gp130-mediated signaling through IL-11 appears to be involved in susceptibility (Table 2).

In contrast with the harmful effect of IL-11 expression during the early course of experimental TB, a human pilot proteome study revealed higher protein levels of IL-11 in fast responders to TB treatment when compared with slow treatment responders [180]. Future studies will therefore be necessary to obtain a more complete picture of the IL-11-mediated immune response and its consequences on the progression of experimental and human TB.

### 3.4. IL-27

In the context of human TB, elevated levels of the IL-12 family cytokine IL-27 are associated with active disease [173]. Both subunits of the heterodimeric cytokine are expressed in human TB granuloma [181] and in TB pleural effusion [182,183], the latter providing the potential to use IL-27 levels as a specific biomarker for the differential diagnosis of tuberculous pleurisy. IL-27 production in TB pleural effusion has been ascribed to various cellular sources including CD4^+^ and CD8^+^ T cells, B cells, NK/NKT cells, monocytes/macrophages and mesothelial cells [183]. IL-27-secreting CD4^+^ T cells were further demonstrated to constitute a terminally differentiated human T cell subpopulation with a distinct expression pattern of transcription factors and pro-inflammatory cytokines [184]. In contrast to these findings, however, gene polymorphisms associated with reduced secretion levels of IL-27 were identified in patients with pulmonary TB [185].

The increased levels of IL-27 during human TB indicate that IL-27 inhibits anti-mycobacterial immunity and thereby contributes to disease progression. In accordance with this hypothesis, IL-27 suppresses protective immune responses during Mtb infection of mice, leading to an impaired mycobacterial containment [17,18,172]. However, at the same time, it also dampens the pathological sequelae of a pathological systemic hyperinflammation [17,18]. Thus, Mtb-infected mice lacking the IL-27Rα chain (IL-27Rα^-/-^) show an elevated release of the pro-inflammatory cytokines TNF and IL-12, resulting in an increased activation of CD4^+^ T cells and amplified macrophage effector functions, which eventually results in significantly reduced bacterial loads [18]. On the other hand, IL-27Rα^-/-^ mice exhibit a hyperinflammatory phenotype during Mtb infection associated with excessive systemic production of pro-inflammatory cytokines, splenomegaly, accelerated cachexia and a decreased survival.

Formation of granuloma represents a hallmark of host defense against Mtb infection [186]. Importantly, TB granuloma in IL-27Rα^-/-^ mice are extremely well-organized, containing a core of activated macrophages surrounded by a layer of lymphocytes [17,18,172]. In addition, during the chronic phase of experimental TB, IL-27Rα-deficient CD4^+^ T cells were found to be superior in assessing the lung parenchyma and associating with an antigen within the lesions [173] (Figure 3C). These T cells exhibit an altered phenotype, as they maintain the expression of the surface molecules programmed death-1 (PD-1), CD69 and CD127, but simultaneously reduce killer cell lectin-like receptor-G1 (KLRG1) expression (Figure 3C). During experimental TB, PD1^+^ CD4^+^ T cells appear to represent a population of self-renewing effector cells, which contribute to anti-mycobacterial immunity, whereas KLRG1^+^ CD4^+^ T cells show all characteristics of terminally differentiated cytokine-secreting cells with a low proliferative potential and a shorter life span [187]. Furthermore, the loss of IL-27Rα-mediated signaling provokes the accumulation of antigen-specific multifunctional CD4^+^ T cells in the lungs of Mtb-infected mice [17]. Multifunctional CD4^+^ T cells are defined by their co-expression of the cytokines IFNγ, TNF and IL-2 and represent a high-quality effector T cell subpopulation [188,189,190] (Figure 3C). During vaccination against Mtb, the superiority of multifunctional T cells for protection has been ascribed, at least partially, to their high cytokine secretion levels on a per cell basis and to their enhanced longevity. Notably, contrary to multifunctional T cells, the overall frequencies of IFNγ-producing CD4^+^ T cells in the lungs of Mtb-infected mice are not affected in the absence of IL-27Rα [17]. Based on these data, it was hypothesized that IL-27 may limit the protective impact of Mtb-specific CD4^+^ T cells at different levels—intrinsically by directly altering the quality and fitness of the induced T cell immune responses and extrinsically by impeding optimal T cell localization within Mtb-containing granulomatous lesions [17,173].

As mentioned before, a further role ascribed to IL-27 comprises its promoting impact on the induction of IL-10-producing regulatory Tr1 cells [131,132] and on the functionality of T_reg_ [128,129]. During experimental TB, T_reg_ infiltrate the site of infection and delay effector T cell migration into the lung during the early course of infection with Mtb [36,37]. Moreover, the production of the immunomodulatory cytokine IL-10 dampens protective immune responses and thereby promotes TB progression in both C57BL/6 and CBA/J mice [191,192]. With regard to the impact of IL-27 on regulatory T cell populations during experimental TB, it was shown that IL-27 expression enhances the accumulation of Tr1 cells in the lung but does not appear to affect numbers of T_reg_ [17] (Figure 3C; Table 2). IL-27-dependent T_reg_, however, have not yet been functionally investigated in the context of TB.

IL-27 is known to suppress the development of IL-17A-producing TH17 cells in mouse models of chronic inflammation [126,127,193,194]. During experimental TB, IL-27Rα^-/-^ mice also exhibit a significantly increased production of IL-17A by TH17 cells [17]. Furthermore, the impact of IL-27 on anti-mycobacterial immunity and TB progression has been connected, at least partially, with the IL-27-mediated suppression of Il-17A production (Figure 3C; Table 2). Thus, in Mtb-infected IL-27Rα^-/-^ mice that additionally lack IL-17A expression, both the increased protection and the exacerbated immunopathology observed in the absence of IL-27Rα are abrogated. While IL-17A appears to mediate neither the suppression of Tr1 cells nor the expression of KLRG1 on CD4^+^ T cells in Mtb-infected IL-27Rα^-/-^ mice, it regulates the accumulation of multifunctional CD4^+^ T cells in the lung. Moreover, the formation of highly structured TB granuloma in the absence of IL-27Rα clearly depends on the production of IL-17A—a function that has been attributed to the cytokine before [30]. In this context, IL-17A also triggers the expression of T cell-attracting chemokines in the lungs of Mtb-infected IL-27Rα^-/-^ mice.

Beyond the capacity of IL-27 to indirectly limit protective cell-mediated immune responses to Mtb through the inhibition of IL-17A production, IL-27-mediated signaling may also directly impair the antimicrobial activity of infected macrophages [126,174] (Figure 3C; Table 2). In human macrophages, IL-27 suppresses phagosomal acidification and phagosome–lysosome fusion by inhibition of vacuolar ATPase (V-ATPase) and lysosomal integrated membrane protein-1 (CD63) and impairment of cathepsin D maturation [126] (Figure 3C). Furthermore, IL-27 induces the autophagy-regulating molecules mTOR and Mcl-1 to suppress IFNγ-mediated autophagy and eventually elimination of intracellular mycobacteria in Mtb-infected human macrophages [174] (Figure 3C; Table 2). In addition to these direct effects on macrophage anti-mycobacterial activity, IL-27 was demonstrated to differentially affect the production of pro- and anti-inflammatory cytokines in macrophages (Figure 3C). Accordingly, IL-27Rα-mediated signaling in murine activated peritoneal macrophages limits the release of IL-12/23p40 and TNF possibly by induction of STAT3 phosphorylation [18] (Table 2). Incubation of Mtb-infected human macrophages with a soluble receptor to neutralize IL-27 (sIL27RA) revealed that IL-27 also antagonizes the activity of IL-18 [195]. Along with IFNγ and TNF, IL-18 plays a pivotal role in reducing mycobacterial growth in human macrophages when IL-12 is supplied and signaling via IL-27 is blocked [175] (Figure 3C; Table 2). On the other hand, in mouse macrophages, IL-27 promotes the transcription of IL-10 through activation of STAT1 and STAT3, which are subsequently recruited to the IL-10 promoter [196] (Figure 3C). Overall, IL-27 can undermine protective immune responses in Mtb-infected macrophages directly by suppressing effector mechanisms and indirectly by restricting the production of pro-inflammatory cytokines. The impaired release of TH1- and TH17-driving cytokines diminishes the subsequent development of a protective T cell response. Future in vivo studies may investigate the overall impact of IL-27Rα-mediated signaling in macrophages on the outcome of Mtb infection.

In summary, during TB, IL-27 represents a “double-edged sword”, as it regulates both protective and immunopathological immune responses [17,18] (Table 2). Both effects can be ascribed to the IL-27-mediated inhibition of IL-17A production [17]. IL-27-dependent immunoregulation appears to impair both the quality and optimal localization of protective T cell responses against Mtb [17,18,173]; however, it may also directly affect the antimicrobial activity of macrophages [174,197].

### 3.5. Other gp130 Cytokines

While, in the context of experimental TB, there are currently only available data in regard to the gp130 cytokines IL-6, IL-11 and IL-27, different human studies indicate the additional contribution of further gp130 cytokines to the TB-associated immunopathology. Secretion levels of the IL-6 cytokine LIF were found to be upregulated in the blood plasma of patients with active and latent TB when compared to non-TB patients [164]. Furthermore, secretion of OSM—which can alternatively signal through the receptor complexes OSMR:gp130 and LIFR:gp130 [55]—appears to be increased in Mtb-infected human monocytes [198]. Together with TNF, the cytokine thereby stimulates the production of the matrix metalloproteinases (MMP)-1 and -3 from human pulmonary fibroblasts. MPPs are involved in the degradation of the extracellular matrix (ECM) [199] and were demonstrated to drive the development of TB cavities in rabbits [200]. Therewith, OSM might contribute to tissue destruction in TB. Finally, the gp130 family-related cytokine IL-31 has been identified as a novel sensitive and specific biomarker for the diagnosis of tuberculous pleurisy [201]. Another recent study further suggests a potential role for IL-31 as a biomarker for the discrimination between patients with latent TB and healthy individuals [202].

Future studies may bring new insights into the possible impact of other gp130 cytokine family members during experimental and human Mtb infection.

## 4. Therapeutical Aspects

### 4.1. Prevention of TB during Therapeutic Targeting of gp130 Cytokines in Autoimmune and Chronic Inflammatory Diseases

The aforementioned specific IL-6/sIL-6Rα trans-signaling inhibitor sgp130Fc represents a promising drug candidate for the management of chronic inflammatory disorders [71]. Under the name olamkicept, the inhibitor is undergoing phase II clinical trials for the treatment of inflammatory bowel disease (IBD) [203] and ulcerative colitis [204]. Investigation of the IL-6/sIL-6Rα *trans*-signaling pathway in the context of TB therefore provides an important contribution to the prevention of TB.

Cytokine-targeting therapies have been successfully used in the management of numerous chronic inflammatory diseases ever since TNF-neutralizing antibodies were deployed to treat rheumatoid arthritis (RA) in the 1990s [205]. However, continuous therapy with such immunomodulatory agents carries the threat of promoting susceptibility to severe bacterial infections. Accordingly, during experimental TB, TNF antagonist-treated mice are highly susceptible to Mtb infection, accompanied by impaired macrophage activation, an unorganized granulomatous response and necrosis development [169,206,207]. In individuals with latent TB, treatment of autoimmune and chronic inflammatory diseases with TNF antagonists is linked to an increased risk of reactivated Mtb infection [6,208,209,210]. Depending on the clinical setting, under TNF antagonist therapy, the relative risk of TB can be increased up to 40 times [6].

In accordance with the pathogenic role of IL-6 in autoimmune and chronic inflammatory diseases such as RA and juvenile idiopathic arthritis [211,212], the IL-6 signaling cascade has been targeted in numerous inflammatory diseases [213]. Several of those anti-IL-6 therapeutics have already been approved or are currently being evaluated in clinical trials. Most accurately described is the mIL-6Rα-neutralizing antibody tocilizumab, which is, inter alia, approved for the treatment of RA and juvenile inflammatory arthritis in multiple countries [71]. Although it appears that, in contrast to inhibition of TNF, blockade of mIL-6Rα does not account for TB progression in mice [214], human studies indicate that treatment with tocilizumab increases the risk of serious infections to a similar extent as TNF antagonists [215]. A meta-analysis published by Schiff and colleagues in 2011 reported enhanced rates of opportunistic infections including TB as well as infections with nontuberculous mycobacteria in patients receiving tocilizumab when compared to a placebo-treated control group [216]. In line with this study, real-world data gathered in Japan suggest that the risk for development of TB during treatment with tocilizumab is comparable to the infection risk during TNF antagonist therapy [217,218]. Even though the discrepancy between these findings and the aforementioned data obtained in mice [214] might result from the choice of mouse model as well as the experimental design, future clinical studies may be necessary to further elucidate the role of the drug in the predisposition to serious infections such as TB.

Tocilizumab, similar to the other IL-6- or mIL-6Rα-neutralizing drugs, blocks both IL-6 *cis*- and IL-6/sIL-6Rα *trans*-signaling [213,219]. Therewith, host-protective activities of IL-6 are suppressed during therapy in the same manner as chronic inflammation. In contrast, the specific blockade of the IL-6/sIL-6Rα *trans*-signaling pathway, which is achieved by treatment with sgp130Fc, still allows non-blocked IL-6 to support productive immune responses in defense against bacterial infections. This may especially include the hepatic acute phase response at the level of innate immunity but also TH1 and TH17 adaptive immune responses [169,213]. Therapeutic targeting of IL-6/sIL-6Rα *trans*-signaling during the treatment of chronic inflammatory diseases hence offers the potential to imply a reduced risk for opportunistic infections. With regard to TB, the previously described mouse experimental data demonstrating that the specific blockade of IL-6/sIL-6Rα *trans*-signaling does not impair protective immunity during Mtb infection support this hypothesis [169]. Moreover, in other animal models of disease, blockade of the *trans*-signaling pathway also appears to be superior when compared to a global inhibition of IL-6-mediated signaling. These include, inter alia, murine models for polymicrobial sepsis [99], bone fracture healing [220] and experimental infection with *Listeria monocytogenes* [221]. However, it still remains to be seen whether the clinical use of the IL-6/sIL-6Rα trans-signaling inhibitor olamkicept may be connected with any increase in the risk of TB reactivation. Together, the introduction of sgp130Fc therapeutics for the treatment of IL-6-driven chronic inflammatory diseases may contribute to TB prevention by providing a lower risk of disease reactivation as one of the major adverse effects of cytokine-directed immunosuppressive therapy.

### 4.2. Potential of Targeting gp130 Cytokines during Treatment of TB

As outlined in several review articles, in the context of novel therapeutic approaches for the treatment of TB, HDTs play an increasingly important role [8,222,223,224,225]. During this type of therapy, administration of antibiotics is combined with immunomodulatory drugs, particularly to reduce the duration of treatment. A number of different candidate host-directed therapeutics against TB are currently undergoing preclinical or clinical trials [222]. These therapeutics target different host pathways to optimize autophagy and phagosomal killing of Mtb within macrophages, immunometabolism, granuloma structure and T cell immunity, but they also aim to dampen exacerbated inflammation [222,224]. One important part of the present HDT approaches comprises the modulation of cytokine signaling either to activate anti-mycobacterial effector mechanisms in macrophages and shape protective T cell immunity or to modulate excessive inflammation.

Antagonists of the gp130 family cytokines, as the present review demonstrates, may also constitute promising candidates for HDT against TB, although no clinical data are yet available on this matter. Unfortunately, in this context, the only currently clinically available drugs are the mIL-6Rα-neutralizing antibodies tocilizumab and siltuximab. Although, in regard to their immunosuppressive properties, both therapeutics are mentioned as potential host-directed anti-TB drugs [222], the aforementioned findings on the risk of treatment with tocilizumab for the development of TB [216,217,218] might argue against this application of mIL-6Rα-neutralizing drugs. However, it should be considered that in connection with a simultaneous antibiotic treatment during adjunct therapy, the administration of cytokine-directed anti-inflammatory drugs may nonetheless have a beneficial effect, as demonstrated by usage of the soluble TNF receptor etanercept during the treatment of HIV-associated TB [226]. In this context, adjunct treatment has a positive therapeutic impact, possibly by disrupting mycobacterial containment within granuloma and eventually promoting the elimination of metabolically active bacteria [222,227]. Importantly, IL-11 as well as IL-27 antagonist treatment may also represent exciting future perspectives for improving therapy for TB. In this regard, IL-11 with a W147A substitution [171] appears to be a candidate antagonist for blocking IL-11 signaling during antibiotic treatment. Accordingly, in Mtb–infected I/St mice, as described above, administration of W147A indeed results in attenuated inflammation in the lung along with an increased survival time of the animals [171]. Since a soluble form of IL-27Rα—sIL-27RA—has been described to naturally control IL-27 receptor binding [228] and treatment with recombinant sIL-27RA has a beneficial effect in septic peritonitis [229], adjunct treatment with sIL-27RA also constitutes an interesting future option to improve the therapy for TB. The inhibitor, however, has not yet been investigated in the context of experimental TB. Nevertheless, it has to be remarked that, whereas the overall knowledge of the IL-11-driven immune mechanisms during Mtb infection is still at the beginning, IL-27 apparently provides a broad spectrum of regulatory functions in the context of TB, underlining the therapeutic potential of interference with IL-27-mediated signaling. However, as IL-27 also prevents immunopathology induced by excessive production of IL-17A [17], inhibition of IL-27-mediated signaling during adjunct TB therapy would have to be strictly controlled.

## 5. Conclusions

The gp130 cytokines IL-6, IL-11 and IL-27 differentially affect the outcome of infection with Mtb. The cytokines thereby exert pleiotropic effects on myeloid and lymphoid cells and modulate pro- and anti-inflammatory immune responses. In particular, the impact of the respective cytokines on IL-17A-producing TH17 cells appears to correlate with disease outcome—whereas, in the absence of IL-6-mediated signaling, both bacterial growth and TH17 immune response are largely unaffected [139] (submitted), IL-27Rα deficiency leads to improved anti-mycobacterial protection by enhanced production of IL-17A [17]. To further revise this connection, however, a potential role of IL-11 in the induction of TH17 cells during TB would also need to be examined. The differential roles of the gp130 cytokines in TB provoke varying potential therapeutic implementations.

## Figures and Tables

**Table 1:** Cytokines and receptor complexes of the gp130 family.

Cytokine	Receptor Complex	Reference ^1^
IL-6	IL-6Rα:gp130:gp130	[50,51]
IL-11	IL11Rα:gp130:gp130	[45]
IL-27 (p28/EBI3)	IL27Rα (WSX-1):gp130	[48]
IL-35 (p35/EBI3)	IL-12Rβ2:gp130	[40]
	gp130:gp130	
IL-39 (p19/EBI3)	IL-23R:gp130	[53]
LIF	LIFR:gp130	[42]
OSM	OSMR:gp130	[46]
	LIFR:gp130	
CNTF	LIFR:CNTFRα:gp130	[44]
	LIFR:IL-6Rα:gp130	
	LIFR:sIL-6Rα:gp130	
CT-1	LIFR:gp130	[47,49]
	LIFR:gp190:gp130	
CLC	LIFR:mCNTFRα:gp130	[43]
NP	LIFR:CNTFRα:gp130	[41]

^1^ determined by different methodologies.

**Table 2:** The role of gp130 in experimental TB ^1^.

	Effect ^2^	Protection ^3^	Pathology ^4^
IL-6	→ inflammation [139]	→ [139]	→ [139]
	↓ macrophages [82,142,143]		
IL-6/sIL-6Rα	→ inflammation [169]	→ [93]	→ [93]
IL-11	↑ inflammation [170,171]	↓ [170,171]	↑ [170,171]
	↑ macrophages [171]		
	↑ neutrophils [170,171]		
IL-27	↓ inflammation [17,18,172]	↓ [17,18,172]	↓ [17,18,172]
	↓ CD4 [17,173]		
	↑ Tr1 [17]		
	↓ TH17 [17]		
	↓ macrophages [18,174,175]		

^1^ determined after aerosol infection of experimental mice; ^2^ pro- and anti-inflammatory functions measured by the overall cytokine response or the cell type-specific effect in lungs; ^3^ protection as determined by counts of colony-forming units in lungs; ^4^ pathology as determined by survival or body weight changes; ↑, increasing effect; ↓, decreasing effect; →, no effect.
